# Identification, expression profiles, and binding properties of chemosensory protein 18 in *Plutella xylostella* (Lepidoptera: Plutellidae)

**DOI:** 10.1093/jisesa/ieae002

**Published:** 2024-01-31

**Authors:** Xingtao Qie, Xizhong Yan, Han Wang, Fangyuan Li, Liming Hu, Chi Hao, Li Ma

**Affiliations:** Department of Plant Protection, College of Plant Protection, Shanxi Agricultural University, Taigu, Shanxi 030801, China; Department of Plant Protection, College of Plant Protection, Shanxi Agricultural University, Taigu, Shanxi 030801, China; Department of Plant Protection, College of Plant Protection, Shanxi Agricultural University, Taigu, Shanxi 030801, China; Department of Plant Protection, College of Plant Protection, Shanxi Agricultural University, Taigu, Shanxi 030801, China; Department of Plant Protection, Institute of Plant Health, ZhongKai University of Agriculture and Engineering, Guangzhou, Guangdong 510225, China; Department of Plant Protection, College of Plant Protection, Shanxi Agricultural University, Taigu, Shanxi 030801, China; Department of Plant Protection, College of Plant Protection, Shanxi Agricultural University, Taigu, Shanxi 030801, China

**Keywords:** *Plutella xylostella* L, olfactory system, chemosensory protein, host volatile, binding ability

## Abstract

Chemosensory proteins (CSPs) are highly efficient carry tools to bind and deliver hydrophobic compounds, which play an important role in the chemosensory process in insects. The diamondback moth, *Plutella xylostella* L. (Lepidoptera: Plutellidae), is a cosmopolitan pest that attacks cruciferous crops. However, the detailed physiological functions of CSPs in *P. xylostella* remain limited to date. Here, we identified a typical CSP, named *Pxyl*CSP18, in *P. xylostella* and investigated its expression patterns and binding properties of volatiles. *PxylCSP18* was highly expressed in antennae and head (without antennae), and the expression level in the male antennae of *P. xylostella* was obviously higher than that in the female antennae. Moreover, *Pxyl*CSP18 has a relatively broad binding spectrum. Fluorescence competitive binding assays showed that *Pxyl*CSP18 had strong binding abilities with 14 plant volatiles (Ki < 10 μM) that were repellent or attractive to *P. xylostella*. Notably, *Pxyl*CSP18 had no significant binding affinity to (Z)-11-hexadecenal, (Z)-11-hexadecenyl acetate, and (Z)-11-hexadecenyl alcolol, which are the pheromone components of *P. xylostella*. The attractive effects of trans-2-hexen-1-ol and isopropyl isothiocyanate to male adults and the attractive effects of isopropyl isothiocyanate and the repellent effects of linalool to female adults were significantly decreased after knocked down the expression of *PxylCSP18*. Our results revealed that *Pxyl*CSP18 might play an important role in host plant detection, avoidance of unsuitable hosts, and selection of oviposition sites; however, it does not participate in mating behavior. Overall, these results extended our knowledge on the CSP-related functions, which provided insightful information about CSP-targeted insecticides.

## Graphical Abstract

**Graphical Abstract:**
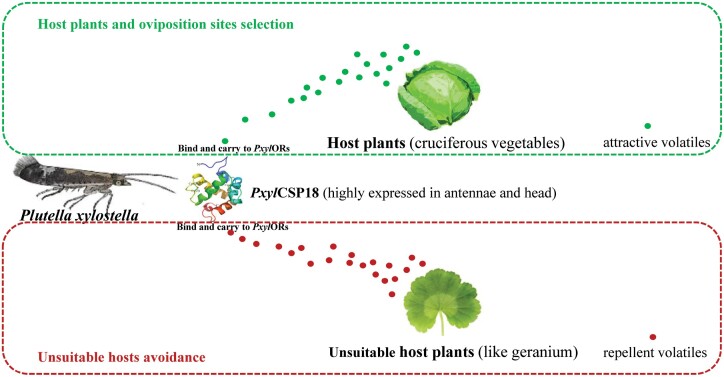

## Introduction

The olfactory system regulates many insect behaviors, including food sources detection, unsuitable host avoidance, selection of oviposition sites, mate choice, and natural enemy avoidance, and plays a pivotal role in the life cycle of insects (Pickett and Woodcock 1996, Field et al. 2000, Riffell and Alarcon 2013, Cao et al. 2020, Wang et al. 2022). As one of the most important olfactory organs, the antennae was covered with many olfactory sensilla that have olfactory sensory neurons (OSNs) (Su et al. 2009, Yan et al. 2017). Volatiles from the environment were received by the olfactory organs, then solubilized and carried by odorant binding proteins (OBPs) or chemosensory proteins (CSPs) to ORNs, and finally, the odor information is transformed into neuronal electrical signals (Pelosi et al. 2006, 2018, Leal 2013). In this process, a wealth of olfaction-related genes were reported to participate in the molecular network of olfaction, such as CSPs, OBPs, odorant-degrading enzymes (ODEs), ionotropic receptors (IRs), olfactory receptors (ORs), sensory neuron membrane proteins (SNMPs), and odorant receptor coreceptor (Orco) (Vogt 2003, Pelosi et al. 2006, 2018, Vogt et al. 2009, Abuin et al. 2011, Leal 2013, Ferguson et al. 2021, Konopka et al. 2021).

Insect CSPs, which have low molecular weight with about 100–120 amino acid residues, are characterized by small size and compact structure that evolutionarily conserved (Pelosi et al. 2006, Fu et al. 2020). The first CSP of insects, OS-D protein, was identified in the antennae of *Drosophila melanogaster* through subtractive hybridization (McKenna et al. 1994). CSPs were initially reported to be involved in limb regeneration, and later were their function as olfactory proteins (Yi et al. 2014). To date, the information available for CSPs, also known as sensory appendage proteins (SAPs), indicated that these small soluble proteins involved in diverse activities and endowed with heterogeneous functions, such as serving as carrier proteins for binding and delivering hydrophobic compounds in chemosensory process (Robertson et al. 1999, Pelosi et al. 2006, 2018, Fu et al. 2020). Studies on the molecular structures suggested CSPs are constituted by 4 conserved cysteines, which arrangement mode shown as Cys_1_-X6-Cys_2_-X18-Cys_3_-X2-Cys_4_, and linked by disulfide bridges between 2 adjacent cysteine residues that resulted in the formation of 2 small loops (Angeli et al. 1999, Zhu et al. 2016, Pelosi et al. 2018, Zeng et al. 2019, Li et al. 2022). Research on the binding abilities showed that CSPs have good affinities with many chemicals, which later was identified that the 2 formed disulfide bridges do not place constraints on the scaffolding of the CSPs, so the proteins can enlarge their binding cavities and adapt to binding different components (Yi et al. 2014, Pelosi et al. 2018). The expression of CSPs was found to be mainly in the antennal; however, they also expressed in nonolfactory organs, which suggested CSPs in connection with different physical functions that are unrelated to chemoreception in insects (Pelosi et al. 2006, Gong, et al. 2007, Gu et al. 2012, Zhu et al. 2016, Martin-Blazquez et al. 2017, Fu et al. 2020).

The diamondback moth (DBM), *Plutella xylostella* L., is a destructive pest of cruciferous vegetables worldwide and causes enormous economic losses annually (Furlong et al. 2013). *Plutella xylostella* relies on the olfactory system to percept the volatile compounds released by host plants to select food sources and oviposition sites (Pivnick et al. 1994). Based on the antennal transcriptome analysis, 118 olfactory genes belonging to 6 chemoreception gene families were identified in *P. xylostella* L., including 54 ORs, 24 OBPs, 15 CSPs, 2 SNMPs, 7 gustatory receptors (GRs), and 16 IRs (Yang, et al. 2017). Furthermore, other 15 putative OBP genes were identified from its genome and transcriptome sequences of *P. xylostella* (Cai et al. 2021). Other 2 putative CSPs were identified from our previous unpublished data. However, only the functions of *Pxyl*CSP1 and *Pxyl*CSP11 have been reported among the 17 identified CSPs (Liu et al. 2010, Yi et al. 2014, Fu et al. 2020). Previous study showed that *Pxyl*CSP1 has a remarkable ability to bind the oviposition inhibitor, Rhodojaponin-III (R-III) (Liu et al. 2010). *Pxyl*CSP11, which mainly expressed in the antennae, has good affinities with special volatiles of cruciferous vegetables, phenethyl isothiocyanate and allyl isothiocyanate, the sex pheromone component, (Z)-11-hexadecenyl acetate, and other volatiles like α-terpineol, 2,4-dimethylheptane, α-terpinene, and phenethyl alcohol (Fu et al. 2020). Hence, we reasoned that *Pxyl*CSP11 also plays an essential role in feeding, mating, and oviposition sites selection. However, the detailed functional characteristics of other CSPs in *P. xylostella* still remain elusive so far.

Herein, we identified a new CSP of *P. xylostella*, *Pxyl*CSP18*. The potential role in chemoreception and host plant recognition in P. xylostella* L will be revealed and characterized. The findings of the study will be of great significance in enhancing our understanding of the olfactory system of *P. xylostella*.

## Materials and Methods

### *Plutella xylostella* Rearing

The larvae and pupae of *P. xylostella* were collected and reared in the Insect Neurobehavioral and Sensory Biology Laboratory, in Shanxi Agricultural University, Taigu Campus. The *P. xylostella* was maintained in a growth chamber at 25 ± 1 °C and 70 ± 5% relative humidity (RH) under a 14-h light (L): 10-h dark (D) photoperiod. *P. xylostella* larvae were fed with fresh cabbage (*Brassica oleracea* L.) and adults were supplied nutrition on absorbent cotton soaked with 10% honey solution.

### Protein Sequence, Domain Identification, Phylogenetic Analysis, and Tertiary Structure Prediction

The amino acid sequences of the *Pxyl*CSP18 were retrieved from the NCBI database (https://www.ncbi.nlm.nih.gov/). Domains were predicted by searching InterPro (http://www.ebi.ac.uk/interpro/search/sequence/) and the annotation of the amino acid sequence in the NCBI database. Homologs of *Pxyl*CSP18 were searched using the BlastP program in the NCBI database. The phylogram was built by the neighbor-joining method using MEGA 5.10 after aligning the selected CSP protein sequences using ClustalW. The tertiary structure of *Pxyl*CSP18 was developed by homology modeling (Swiss-Model: https://swissmodel.expasy.org/) based on the CSPsg4 of locust *Schistocerca gregaria* (Forskáhl) (Orthoptera: Acrididae).

### RNA Extraction, cDNA Synthesis, and Quantitative Real-Time PCR

The total RNA of different tissue samples was isolated using TriPure Isolation Reagent (Roche, Switzerland) and purified by Direct-zol RNA Miniprep (Zymo Research, USA) according to the manufacturer’s instructions. The extracted total RNA was quantified by spectrophotometric analysis, and the integrity was verified by agarose gel electrophoresis. cDNA was synthesized from the purified total RNA using Transcriptor First cDNA Synthesis Kit (Roche, Switzerland).

The quantitative real-time PCR (qRT-PCR) assay of *PxylCSP18* was performed on a Bio-Rad CFX Connect Real-Time Detection System (Bio-Rad, California, USA) using the 2 × SG Fast qPCR Master Mix (Sangon, China). The gene of ribosomal protein S4 (*Rps4*) was used as an endogenous control (Fu et al. 2020). The results were evaluated using a relative quantitative method (2^−ΔΔCt^). All analyses were performed with 3 biological replicates. The primers used in qRT-PCR are listed in Table 1. The *R*^2^ values of the standard curves were over 0.980 and the calculated amplification efficiency was 90%–110%. These indicated that the qRT-PCR were done in optimal conditions.

**Table 1.** Primers used in the study

Primers	Sequence (5ʹ–3ʹ)
Quantitative RT-PCR	
Q-*PxylCSP18*-F	GATGCTAAGGATTTCAAGAAGGTAA
Q*-PxylCSP18*-R	CAACTCTCAGGGTGTCTCTCTCTT
Q*-Rps4*-F	ATGGATGTTGTGTCGATTGAAAAGA
Q*-Rps4*-R	GGGGTTGCCCAGGTCAGAT
*PxylCSP18* gene cloning	
*PxylCSP18*-F	CGCGATATCGATTTCTACAGTAGTCGGTATGACA
*PxylCSP18*-R	CCGCTCGAGTTAGTCATCTTGTAGCAGGAATTTA
dsRNA synthesis	
** **dsCSP18-F	CAAACCGTGTGTGGAAAATG^a^
** **dsCSP18-R	CTAGTTGTAACAAAAAGGTGC^a^
** **dsGFP-F	GTGTTCAATGCTTTTCCCGT^a^
** **dsGFP-R	CAATGTTGTGGCGAATTTTG^a^

Underline showed the *EcoR* V and *Xho* I restriction enzyme sites.

^a^Only gene-specific parts of the primers are listed. These are preceded by the T7 adaptor TAATACGACTCACTATAGGG for dsRNA synthesis.

All data were analyzed through a 1-way analysis of variance (ANOVA) followed by Tukey’s honest significant difference (HSD) test (*P* < 0.05) in SPSS Statistics 17.0 software (SPSS Inc., Chicago, IL).

### Double-Stranded RNA Synthesis and RNA Interference

The *PxylCSP18* target fragment for RNA interference (RNAi) was amplified by PCR using the synthesized cDNA as template, and the primers were listed in Table 1. Both ends of the target fragment were added with T7 promoter sequences. After ensuring the bands of the PCR products were at the correct positions on the agarose gel, the specificity of the fragments was further confirmed via sequencing. The PCR products were purified using the Gel Extraction Kit (Promega, Madison, WI). The purified products were used to synthesize double-stranded RNA (dsRNA) with the T7 RiboMAX TM Express RNAi System (Promega, Madison, WI) according to the manufacturer. The purified dsRNA was quantified using spectrophotometry analysis, and the purity and integrity of dsRNA were verified using agarose gel electrophoresis. Finally, the purified dsRNA was then stored at −80 °C. The DNA fragment for the synthesis of ds*GFP* was amplified by PCR using our previously constructed reporter plasmid containing the open reading frame of GFP as a template (Ma et al. 2020), and the primers were listed in Table 1. ds*GFP* was also synthesized with the purified PCR products, as described above, to serve as an RNAi control.

The pupae the day before eclosion were collected and used for RNA interference. The pupae were anesthetized on ice and then injected with 1 μl of dsRNA (10 μg/μl) at the dorsal site of the abdomen using a Nanoject III micro-injector (Drummond Scientific, Broomall, PA) equipped with glass capillaries prepared using a P-97 micropipette puller (Sutter Instrument Co., Novato, CA). The antennae of day-2 unmated female and male adults were collected to detect the efficiency of RNAi-mediated knockdown of *PxylCSP18* through qRT-RNA. Three independent biological replicates were performed.

### Expression and Purification of Recombinant PxylCSP18

The coding region of mature *Pxyl*CSP18 was cloned from the synthesized cDNA with the specific primers, in which an *EcoR* V and an *Xho* I restriction sites were introduced (Table 1). The amplified product was cloned into the pET-32a vector (Novagen, USA), followed by transformation into the competent *E. coli* BL21 (DE3) (Tiangen, China). The expression of *Pxyl*CSP18 in the bacteria was induced by 0.6 mM isopropyl-beta-d-thiogalactopyranoside (IPTG) overnight. Subsequently, the bacterial cells were collected through centrifugation and resuspended into phosphate-buffered saline (PBS) solution, then homogenized through sonication on ice using an ultrasonic processor (Sonics Vibra-Cell, USA). *Pxyl*CSP18 was purified from the soluble faction using a Ni^2+^-NTA column (Roche, Switzerland) through a stepwise elution with 20, 50, 100, 150, and 500 mM imidazole in PBS. The fractions containing purified *Pxyl*CSP18 were collected and dialyzed against 20 mM Tris–HCl buffer containing 50 mM NaCl and 2 mM CaCl_2_ (pH 7.4) overnight, followed by digestion by recombinant enterokinase (Solarbio, China) for 12 h to cleave the fragment containing Trx, His, and S tags, which led to *Pxyl*CSP18 lose the affinity for the Ni^2+^-NTA column. After repurified on Ni^2+^-NTA column, highly purified recombinant *Pxyl*CSP18 was acquired, which was then dialyzed against 20 mM Tris-HCl (pH 7.4). Finally, the *Pxyl*CSP18 was concentrated through ultrafiltration using a centrifugal device (3 kDa, Merck Millipore, USA), and the concentration was determined using the BCA Protein Assay Kit (Sangon, China). The purified recombinant *Pxyl*CSP18 was verified by peptide mass fingerprinting (Sangon Biotech, China).

### Fluorescence Competitive Binding Assays

The fluorescence competitive binding assays were performed as described previously (Fu et al. 2020, Hua et al. 2021) with some modifications. Briefly, the emission spectra of the fluorescent probe, N-phenyl-1-naphthylamine (1-NPN, Sigma-Aldrich, USA), were recorded from 350 to 600 nm with 5 nm width of the emission slits and the excitation wavelength was 337 nm. To measure the affinity of 1-NPN to *Pxyl*CSP18, 1 µM protein in 20 mM Tris–HCl buffer (pH 7.4) was titrated with 1-NPN to final concentrations ranging from 1 to 17 µM in 1 cm light path quartz cuvette. After incubation for 3 min, the fluorescence intensity for each test was detected on an RF-5301 fluorescence spectrophotometer (Shimadzu, Japan). The average data of the recorded fluorescence intensity at the maximum emission wavelength were linearized using the Scatchard equation, and the dissociation constants for 1-NPN were then calculated (Sideris et al. 1992, Hua et al. 2021). Sixteen ligands, including 3 sex pheromone components ((Z)-11-hexadecenal, (Z)-11-hexadecenyl acetate, and (Z)-11-hexadecenyl alcolol) and some host volatiles (terpene, ketone, aldehyde, alcohol, and ester volatiles), were detected the affinities of *Pxyl*CSP18. The final concentrations for each ligand ranging from 1 to 17 µM were added into the mixed solution of 1-NPN probe (1 µM) and *Pxyl*CSP18 (1 µM). The acquired data were calculated from the Scatchard plots for dissociation constants (K_d_) and the curves, assuming *Pxyl*CSP18 was active completely and bound to the ligands in a 1:1 ratio at saturation.

K_d_ of the competitor was calculated according to the following equation: Ki = [IC_50_]/(1 + [1-NPN]/K_1-NPN_), where [IC_50_] is the concentration of ligand halving the initial value of fluorescence, [1-NPN] is the free concentration of 1-NPN and, K_1-NPN_ is the dissociation constant of the protein/1-NPN complex (Hua et al. 2021).

### Y-Tube Olfactometer Bioassays

The responses of the day-2 unmated female and male adults to the volatile compounds were performed using a glass Y-tube olfactometer with a central glass tube (5 cm long × 2.5 cm ID) and 2 lateral glass arms (15 cm long × 2.5 cm ID) with a 75° inside angle as described previously (Yan et al. 2023), with some modifications. Before bioassays, all glasswares were cleaned and washed with absolute alcohol and dried at 100 °C for 5 min. A moistened and activated charcoal-filtered airflow (350 mL/min) was pumped into each source container using an atmospheric sampling instrument (QC-1S, Beijing Institute of Labor Protection Science, Beijing, China). The behavioral response of male and female adults of *P. xylostella* to trans-2-hexen-1-ol, isopropyl isothiocyanate, myrcene, linalool were then observed. Each component was diluted with paraffin oil to 10 μg/μl. For each chemical, an aliquot of 10 μl was applied to a strip of filter paper (0.5 × 5 cm) and placed in 1 source container. A filter paper strip loaded with the same volume of paraffin oil and placed in the second source container was used as a control. Each test male or female moth was released at the entrance of the central glass tube of the Y-tube olfactometer and observed for 5 min. A moth was considered to have made a “choice” when it moved more than 10 cm into either arm and remained there for at least 30 s; “no choice” was recorded when the moth did not make a choice within 5 min. The positions of the arms were reversed after 5 insects to avoid positional bias. The experimental device was cleaned after every 10 insects by washing it with absolute acetone and followed by heating at 100 °C for 5 min. Each concentration of compound stimulus was tested with 30 insects, which successfully made a “choice.” The choice index was calculated as the number of responses of moths to control arms divided by the total number of test moths.

## Results

### Sequence, Tertiary Structure, and Phylogenetic Analyses of *Pxyl*CSP18

**Fig. 1.**
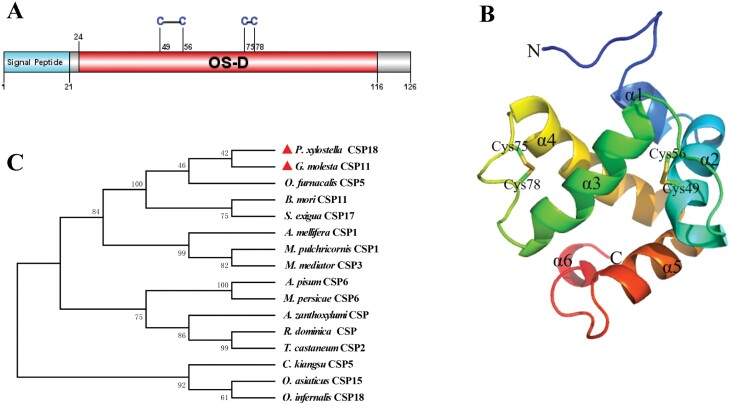
The A) functional domain, B) tertiary structure, and C) phylogenetic analyses of *Pxyl*CSP18. A) A predicted signal peptide that consists of the first 21 residues and an OS-D domain (24-116 amino acid residues) was indicated in the amino acid sequence of *Pxyl*CSP18 (XP_011563205.3). The 4 conserved cysteines (Cys49, Cys56, Cys75, and Cys78), which were shown as Cys_1_-X6-Cys_2_-X18-Cys_3_-X2-Cys_4_, were located at the OS-D domain. B) *Pxyl*CSP18 was composed of 6 alpha helixes: α1 (Ile33-Glu38), α2 (Asp40-Asp52), α3 (Asp58-Gln72), α4 (Pro80-Arg96), α5 (Pro98-Tyr108), and α6 (Arg115-Phe118). A and B) Two disulfide bridges were formed by Cys49-Cys56 and Cys75-Cys78. C) The phylogram was built by the neighbor-joining method using MEGA 5.10 after aligning the selected CSP protein sequences using ClustalW. The numbers on the branches represent the values of bootstrap. *Grapholita molesta* CSP11 (*G. molesta* CSP11): ALC79597; *Ostrinia furnacalis* CSP5 (*O. furnacalis* CSP5): BAV56809.1; *Bombyx mori* CSP11 (*B. mori* CSP11): XP_012549404; *Spodoptera exigua* CSP17 (*S. exigua* CSP17): AVC68636.1; *Apis mellifera* CSP1 (*A. mellifera* CSP1): NP_001071288.1; *Meteorus pulchricornis* CSP1 (*M. pulchricornis* CSP1): AQN78395.1; *Microplitis mediator* CSP3 (*M. mediator* CSP3): ANT46051.1; *Acyrthosiphon pisum* CSP6 (*A. pisum* CSP6): ULF48247.1; *Myzus persicae* CSP6 (*M. persicae* CSP6): ACJ64047.1; *Agrilus zanthoxylumi* CSP (*A. zanthoxylumi* CSP): QTJ02340.1; *Rhyzopertha dominica* CSP (*R. dominica* CSP): KAI7815634.1; *Tribolium castaneum* CSP2 (*T. castaneum* CSP2): NP_001039277.1; *Ceracris kiangsu* CSP5 (*C. kiangsu* CSP5): QJX74393.1; *Oedaleus asiaticus* CSP15 (*O. asiaticus* CSP15): ATI99854.1; *Oedaleus infernalis* CSP18 (*O. infernalis* CSP18): AYN71367.1.

*PxylCSP18* (GeneID: 105393168) encodes a polypeptide of 126 amino acid residues (XP_011563205.3), with a predicted signal peptide that consists of the first 21 residues (Fig. 1A). The functional domain analysis indicated that *Pxyl*CSP18 contains an OS-D domain (24–116 amino acid residues), which was the typical domain of CSP (Fig. 1A). The tertiary structure prediction showed that *Pxyl*CSP18 was composed of 6 alpha helixes: α1 (Ile33-Glu38), α2 (Asp40-Asp52), α3 (Asp58-Gln72), α4 (Pro80-Arg96), α5 (Pro98-Tyr108), and α6 (Arg115-Phe118) (Fig. 1B). Two disulfide bridges were formed by Cys49-Cys56 and Cys75-Cys78 (Fig. 1A and B). *Pxyl*CSP18 is orthologous to the CSP11 of *Grapholita molesta* (Busck) (Lepidoptera: Tortricidae), with 70% identity in amino acid sequence. Phylogenetic analysis revealed that *Pxyl*CSP18 was in the same clade as *G. molesta* CSP11 and *Ostrinia furnacalis* CSP5 (Fig. 1C). Taken together, these results suggested that the identified *Pxyl*CSP18 was a typical chemosensory protein.

### Expression Pattern of *PxylCSP18*

**Fig. 2.**
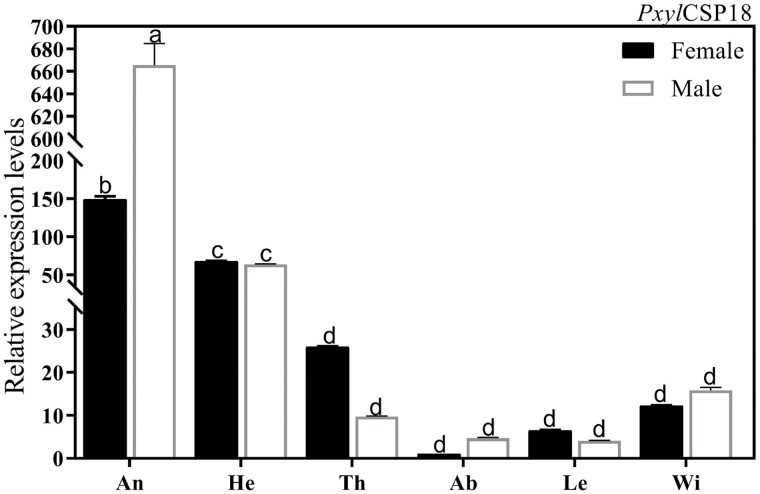
The tissue expression levels of *PxylCSP18* in the female and male *P. xylostella* adults. The tissues included antennae (An), heads without antennae (He), thoraxes (Th), abdomens (Ab), legs (Le), and wings of the female and male adults. The values shown were the mean (±SEM) of 3 independent experiments. Lowercase letters (a, b, c, d) above the error bars denoted significant differences in the tissues at *P* < 0.05. *P* values were determined by ANOVA followed by Tukey’s honestly significant difference test.

The expression levels of *PxylCSP18* in different tissues (antennae, heads without antennae, thoraxes, abdomen, legs, and wings) of female and male adult moths were analyzed by qRT-PCR. The results showed that *PxylCSP18* was expressed in all the tested tissues of females and males, and the highest expression level was found in antennae (Fig. 2, *F*_(11, 24)_ = 1087, *P* < 0.001). The expression level of *PxylCSP18* in male antennae was obviously higher than that in female antennae (Fig. 2). Additionally, the expression of *PxylCSP18* in heads (without antennae) was higher than that in other tissues, but it was much lower than that in antennae, which suggested that *Pxyl*CSP18 mainly plays a role in antennae (Fig. 2).

### The Purification of the Recombinant *Pxyl*CSP18

**Fig. 3.**
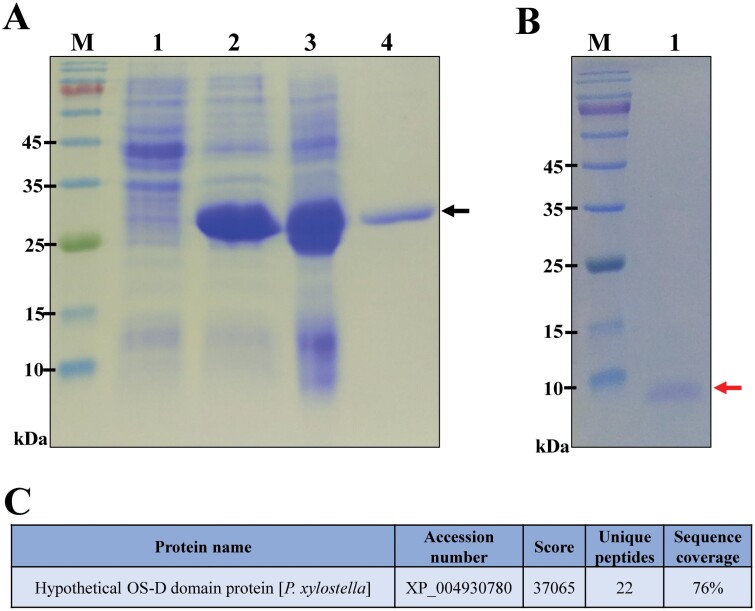
Expression and purification of recombinant *Pxyl*CSP18 analyzed by SDS–PAGE. A) M: protein molecular mass markers. Lane 1: total proteins from uninduced *E. coli* cells; lane 2: total proteins from induced *E. coli* cells; lane 3: soluble proteins from induced *E. coli* cells; lane 4: purified fusion protein before enterokinase digestion (pointed by the black arrow). B) Lane 1: purified *Pxyl*CSP18 (pointed by the black arrow). C) The mass spectrometry results of the protein band excised from the gel in (B).

To further investigate the physiological function of *Pxyl*CSP18, the recombinant *Pxyl*CSP18 was expressed and purified from *E. coli* (DE3). The recombinant *Pxyl*CSP18 was expressed as a fusion protein with a hexahistidine and a Trx tags in the N-terminus to facilitate its correct folding and purification. The induction and purification of recombinant *Pxyl*CSP18 with Trx-hexahistidine tags was illustrated as Fig. 3A. After digestion with enterokinase, the *Pxyl*CSP18 lost the Trx-hexahistidine tags. After purified on Ni^2+^-NTA column, highly purified recombinant *Pxyl*CSP18 without tags was acquired (Fig. 3B). The purified recombinant protein was verified by peptide mass fingerprinting as hypothetical OS-D domain protein, which meant the identified *Pxyl*CSP18 (XP_011563205.3) (Fig. 3C and Supplementary form 1).

### The Binding Affinities of *Pxyl*CSP18 to Ligands

**Fig. 4.**
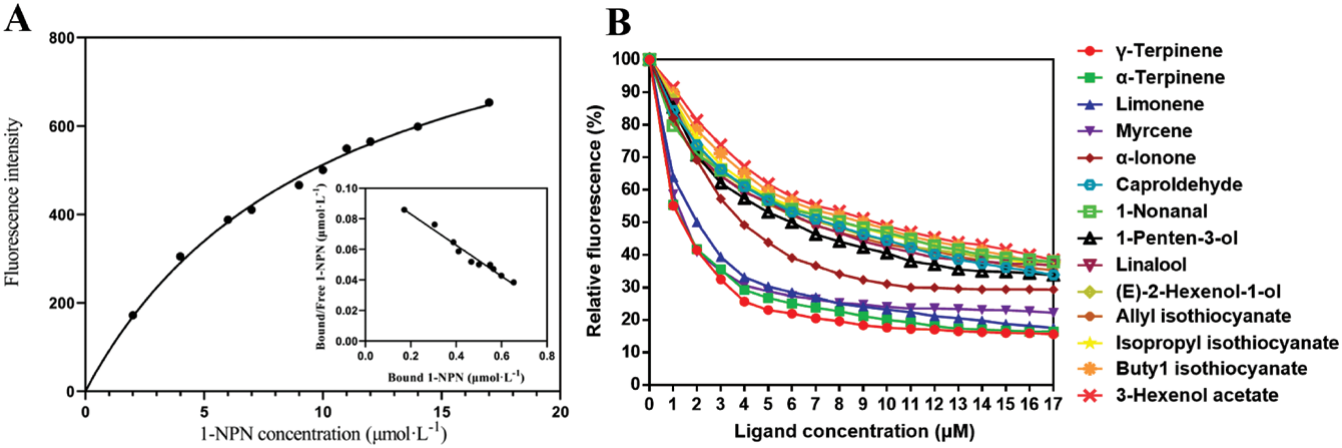
The binding affinities of *Pxyl*CSP18 with various ligands. A) Binding curve of the *Pxyl*CSP18 with fluorescent probe1-NPN and the relative Scatchard plot analysis. B) Competitive binding curves of *Pxyl*CSP18 with various odorant ligands.

The binding affinities of *Pxyl*CSP18 to kinds of ligands were performed with fluorescence competitive binding assays using 1-NPN as the fluorescence probe. The dissociation constant (K_1-NPN_) of 9.85 μM was calculated with the saturated and linear Scatchard plots (Fig. 4A). Then it was used to calculate the values of Ki for individual ligands. Seventeen ligands, including 3 sex pheromone components and 14 plant volatiles were examined for the binding affinities of *Pxyl*CSP18. The competitive binding curves of *Pxyl*CSP18 and various ligands were shown as Fig. 4. *Pxyl*CSP18 exhibited strong binding affinities with the 14 plant volatiles (Ki < 10 μM), including kinds of identified repellent and attractive plant volatiles to *P. xylostella* (Table 2). In the tested volatiles, of which 5 had Ki < 5 μM (γ-terpinene: 0.9698 μM, α-terpinene: 1.0329 μM, myrcene: 1.0711 μM, limonene: 1.4798 μM, α-Ionone: 3.3476 μM) and 9 had 5 μM < Ki < 10 μM (caproldehyde: 5.9808 μM, 1-nonanal: 6.3408 μM, 1-penten-3-ol: 5.7254 μM, linalool: 6.0726 μM, (E)-2-hexenol-1-ol: 6.1141 μM, allyl isothiocyanate: 5.7677 μM, isopropyl isothiocyanate: 6.5212 μM, buty1 isothiocyanate: 7.0712 μM, (Z)-3-hexenol acetate: 7.2540 μM) (Table 2). However, *Pxyl*CSP18 had no significant binding affinity to (Z)-11-hexadecenal, (Z)-11-hexadecenyl acetate, and (Z)-11-hexadecenyl alcolol (Table 2).

**Table 2.** Binding affinities of *Pxyl*CSP18 with volatile ligands

Ligands	CAS number	Company	IC_50_ (μM)	Ki (μM)
Plant volatiles				
Monoterpenes				
γ-Terpinene^b^	99-85-4	Sigma–Aldrich	1.215	0.9698
α-Terpinene^a^	99-86-5	Sigma–Aldrich	1.294	1.0329
Myrcene^b^	123-35-3	Sigma–Aldrich	1.342	1.0711
Limonene	5989-54-8	Sigma–Aldrich	1.854	1.4798
** **Ketones				
α-Ionone	127-41-3	Sigma–Aldrich	4.194	3.3476
Aldehydes				
Caproldehyde^a^	66-25-1	Sigma–Aldrich	7.493	5.9808
1-Nonanal^※^	124-19-6	Sigma–Aldrich	7.944	6.3408
(E)-2-Hexenal-1-ol^a^	928-95-0	Sigma–Aldrich	7.66	6.1141
Alcohols				
1-Penten-3-ol^a^	616-25-1	Sigma–Aldrich	7.173	5.7254
Linalool^b^	78-70-6	Sigma–Aldrich	7.608	6.0726
Esters				
Allyl isothiocyanate^a^	57-06-7	Sigma–Aldrich	7.226	5.7677
Isopropyl isothiocyanate^a^	2253-73-8	Sigma–Aldrich	8.17	6.5212
Buty1 isothiocyanate^a^	592-82-5	Sigma–Aldrich	8.859	7.0712
(Z)-3-Hexenyl acetate^a^	3681-71-8	Sigma–Aldrich	9.088	7.2540
Sex pheromone components from *P. xylostella*				
(Z)-11-hexadecenyl acetate	34010-21-4	Macklin	N.D.	N.D.
(Z)-11-hexadecenal	53939-28-9	Macklin	N.D.	N.D.
(Z)-11-hexadecenyl alcolol	56683-54-6	Macklin	N.D.	N.D.

N.D. is the abbreviation for not detected, which means that no significant binding was detected in the assay.

^a^Means the plant volatiles, which are attractive to *P. xylostella*, are detected in cruciferous vegetables (Reddy and Guerrero 2000a, Han et al. 2001, Dai et al. 2008, Li et al. 2012, Yan et al. 2023).

^b^Means the plant volatiles, which are repellent to *P. xylostella*, are detected in nonhost plant (Song et al. 2022).

**Fig. 5.**
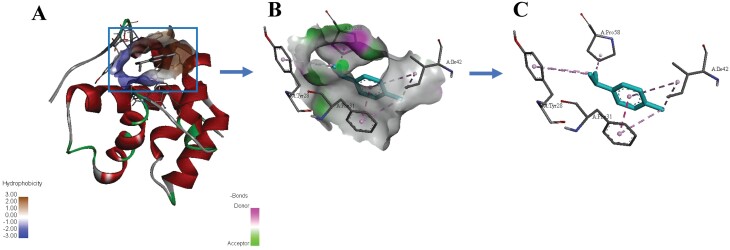
Molecular docking analysis of the binding characters about *Pxyl*CSP18 with the best ligand. A) Three-dimensional view for molecular docking of *Pxyl*CSP18 with γ-terpinene. B) The binding domain of the *Pxyl*CSP18-γ-terpinene. C) The key interactions at the binding domain of the *Pxyl*CSP18-γ-terpinene. Pi-Pi Stacked was formed by γ-terpinene-Phe: 31; Pi-Alkyl or Alkyl bonds were formed by γ-terpinene-Tyr: 28, γ-terpinene-Ile: 42, and γ-terpinene-Pro: 58; van der Waals forces were formed by γ-terpinene-Tyr: 46 and γ-terpinene-Asp: 59.

The molecular docking of *Pxyl*CSP18 and γ-terpinene based on homology modeling was performed to further investigate the combination of *Pxyl*CSP18 and ligands. The *Pxyl*CSP18 had a strong binding affinity to γ-terpinene and the binding free energy of protein-ligand complex was -5.7 kcal/mol. The binding pocket was formed by α1, α2, and α3 and located near the N-term of *Pxyl*CSP18 (Fig. 5A and B). Concretely, Pi-Pi Stacked was formed by γ-terpinene-Phe: 31; Pi-Alkyl or Alkyl bonds were formed by γ-terpinene-Tyr: 28, γ-terpinene-Ile: 42, and γ-terpinene-Pro: 58; van der Waals forces were formed by γ-terpinene-Tyr: 46 and γ-terpinene-Asp: 59 (Fig. 5B and C). These forces were the main forces for *Pxyl*CSP18 and γ-terpinene. Hence, we here demonstrated that *Pxyl*CSP18 had a strong binding ability to many repellent and attractive plant volatiles on different levels (Table 2, Figs. 4B and 5). These data suggested *Pxyl*CSP18 plays an important role in the unsuitable host avoidance and food sources/host detection.

### Behavioral Responses of *P. xylostella* Adults to Volatile Compounds After Knockdown the Expression of *PxylCSP18*

**Fig. 6.**
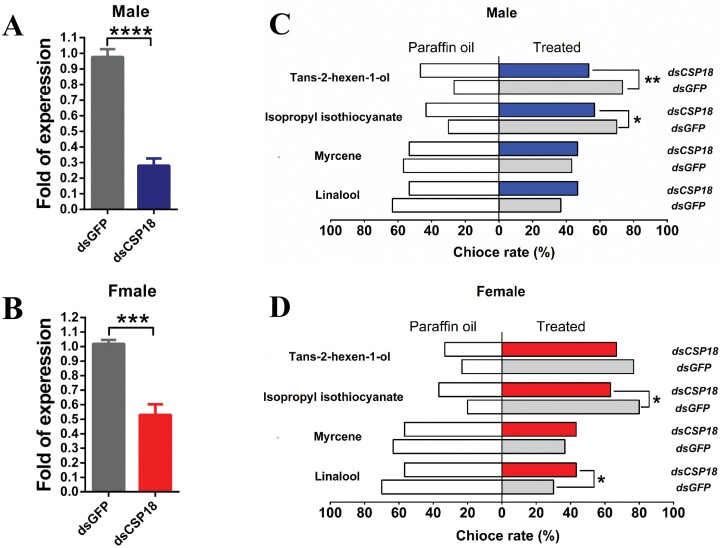
Behavioral responses of *P. xylostella* adults to volatile compounds after knocking down the expression of *PxylCSP18*. The efficiency of RNAi-mediated knockdown of *PxylCSP18* in A) male and B) female adults. Relative expression levels were analyzed by qRT-RNA at the third day after injected ds*CSP18* with the moths injected by dsGFP as control groups. The expressions of *PxylCSP18* were normalized with ribosomal protein S4 gene (rps4) of the *P. xylostella*. For A) and B), the values shown are the mean (±SEM) of 3 independent biological experiments. The statistical differences between the compared groups were denoted with asterisks. *P*-values were determined by student *t* test (2 tailed). ***P < 0.001; ****P < 0.0001. Olfactory responses of C) male and D) female adults of *P. xylostella* to E)-2-hexen-1-ol, isopropyl isothiocyanate, myrcene, linalool at a concentration of 10 μg/μl after knocking down the expression of *PxylCSP18*. *Significant difference at *P* < 0.05. **Significant difference at *P* < 0.01 (χ^2^ test).

After injected ds*CSP18* for 3 days, the expression of *PxylCSP18* decreased by approximately 70% and 48% in the unmated male (Fig. 6A) and female (Fig. 6B) adults (day 2), respectively. Trans-2-hexen-1-ol and isopropyl isothiocyanate were randomly selected as the attractive volatile compounds and myrcene and linalool were randomly chosen as the repellent volatile compounds, to explore the behavioral responses of *P. xylostella* adults to volatile compounds after knocked down the expression of *PxylCSP18*. The attractive effects of trans-2-hexen-1-ol and isopropyl isothiocyanate to *P. xylostella* male adults were significantly decreased after knocked down the expression of *PxylCSP18* (Fig. 6C). The attractive effects of isopropyl isothiocyanate and the repellent effects of linalool to *P. xylostella* female adults were significantly weakened after knocked down the expression of *PxylCSP18* (Fig. 6D), while the repellent effects of myrcene and linalool to *P. xylostella* male adults, and attractive effects of trans-2-hexen-1-ol and repellent effects of myrcene *P. xylostella* female adults decreased inconspicuously after knocked down the expression of *PxylCSP18* (Fig. 6C and D).

## Discussion

CSPs are abundant in the sensillum lymph, and they are mainly responsible for capturing and transporting odor molecules from the external environment to ORs (Pelosi et al. 2006). Thus, molecular function studies on CSPs are of great significance to reveal chemical communication mechanism between insects and host plants.

In this study, the *PxylCSP18* was cloned from *P. xylostella*, and the properties of the *Pxyl*CSP18 were discussed. The *Pxyl*CSP18 has a signal peptide in the N-terminal and 4 highly conserved cysteines in the OS-D domain, which are the typical common characteristics of insect CSPs (Zhou et al. 2006, Zhang and Lei 2015, Ali et al. 2019). The tertiary structure and phylogenetic analyses further confirmed that the *Pxyl*CSP18 was a typical CSP. *Pxyl*CSP18 was clustered in a group with other lepidopterous CSPs, indicating the conservation of phylogeny in insect CSPs. According to antennal transcriptome analysis, *Gmol*CSP11 is highly expressed in female antennae of *G. molesta* (Li et al. 2015). *Pxyl*CSP18 is orthologous to *Gmol*CSP11 with 70% identity in amino acid sequence. Therefore, combined with the result of this study, we speculate that *Pxyl*CSP18 may have functionality similar to *Gmol*CSP11.

The expression profiles of CSPs in different tissues suggest functional differentiation of CSPs (Yang et al. 2014). The analysis of the relative expression in various tissues of *P. xylostella* showed that *PxylCSP18* was mainly expressed in antennae and the expression level in male antennae was obviously higher than that in female antennae. Generally, CSPs were mainly isolated from sensory organs of insect (Pelosi et al. 2006). Antennae and maxillary palps are the main olfactory organs of insect, and they are covered with kinds of porous sensilla (Wicher and Miazzi 2021). *PxylCSP18* was also found to have a high expression in the head (without antennae) of *P. xylostella*, but it was much lower than that in antennae. Therefore, *Pxyl*CSP18 might play a role in other olfactory organs of *P. xylostella*, such as the maxillary palps, except for antennae.

γ-Terpinene and myrcene were repellent to *P. xylostella* (Song et al. 2022). The volatiles of (E)-2-hexenal, (Z)-3-hexenyl acetate, α-terpinene, caproldehyde, 1-Nonanal, 1-penten-2-ol, linalool, allyl isothiocyanate, isopropyl isothiocyanate, and butyl isothiocyanate from cruciferous vegetables were attractive to *P. xylostella* (Reddy and Guerrero 2000a, Han et al. 2001, Dai et al. 2008, Li et al. 2012, Yan et al. 2023). *Pxyl*CSP18 could strongly bind these repellent and attractive plant volatiles. Accordingly, our study demonstrated that *Pxyl*CSP18 has a relatively broad binding spectrum of plant volatiles. *Pxyl*CSP18 might play a crucial role in host plants detection, unsuitable host avoidance, and oviposition sites selection.

The sex pheromone components of *P. xylostella* were identified as (Z)-11-hexadecenal, (Z)-11-hexadecenyl acetate, and (Z)-11-hexadecenyl alcolol with a ratio of 9.4: 100: 17 (unpublished data). Based on the components, sexual attractants for *P. xylostella* have been widely developed and applied (Reddy and Guerrero 2000b, Schroder et al. 2000, Cui and Zhu 2016). Although the expression level of *PxylCSP18* in male antennae was obviously higher than that in female antennae, *Pxyl*CSP18 had no significant binding affinity to (Z)-11-hexadecenal, (Z)-11-hexadecenyl acetate, and (Z)-11-hexadecenyl alcolol, which suggested that *Pxyl*CSP18 might not participating in mating behavior.

Insect CSPs are usually thought to contribute to the transport of ligand from the sensilla cuticle to receptors in olfactory neurons (Yi et al. 2014). Hence, based on the results discussed above, *Pxyl*CSP18 might be involved in the perception of host volatiles and sex pheromones (Zhang et al. 2014, Zeng et al. 2018). Furthermore, the binding characteristics analysis showed that *Pxyl*CSP18 had strong binding affinities with the 14 plant volatiles. Meanwhile, the repellent and attractive effects of the adults to the repellent and attractive volatile compounds were decreased after knockdown of *PxylCSP18*. These results together revealed that *Pxyl*CSP18 play an important role in olfactory perception by binding and transporting plant volatiles (Peng et al. 2017, Hua et al. 2021, Li et al. 2021, Wang et al. 2021).

In conclusion, by discussing the characteristics and functions of *Pxyl*CSP18 in *P. xylostella*, this study provides new information for investigating the physiological role of insect CSPs. It will contribute to understanding the underlying the olfactory communication mechanism of *P. xylostella*.

## Supplementary Material

ieae002_suppl_Supplementary_Material
